# Clinical Outcomes and Safety Assessment of Flexible Ureteroscopy as an Outpatient Procedure: A Retrospective Single-Center Study

**DOI:** 10.3390/life14091131

**Published:** 2024-09-07

**Authors:** George F. Mitroi, Petru Octavian Drăgoescu, Mihaela Roxana Mitroi, George G. Mitroi, Iulia Bianca Dudan, Tudor Cristian Timotei Popescu, Cristian Mihai Nedelcuță, Andrei Ioan Drocaș

**Affiliations:** 1 Department of Urology, Faculty of Medicine, University of Medicine and Pharmacy of Craiova, 200349 Craiova, Romania; george.mitroi@umfcv.ro (G.F.M.);; 2 Department of Otorhinolaryngology, Faculty of Medicine, University of Medicine and Pharmacy of Craiova, 200349 Craiova, Romania; 3 Department of Dermatology, Faculty of Medicine, University of Medicine and Pharmacy of Craiova, 200349 Craiova, Romania; 4 Faculty of Medicine, University of Medicine and Pharmacy of Craiova, 200349 Craiova, Romania

**Keywords:** flexible ureteroscopy, outpatient procedure, safety, complications, urinary stones

## Abstract

Nephrolithiasis, or kidney stone disease, is a significant global health issue in urology, requiring effective management strategies. The management of nephrolithiasis through flexible ureteroscopy (fURS) is increasingly gaining acceptance; however, it is associated with significant costs related to consumables, pharmacotherapy, specialized equipment, and general anesthesia (GA). Limited resources and the need to optimize the cost effectiveness ratio have driven the shift to day-case procedures, offering financial and operational benefits and improving patient satisfaction. This outpatient care approach addresses clinical and economic challenges. For same-day discharge, spinal anesthesia (SA) is essential for fURS, as GA does not permit safe immediate discharge. This retrospective study investigates the feasibility of same-day discharge following fURS procedures performed under SA. Analyzing data from 401 patients who underwent 414 fURS procedures between January 2020 and December 2023, this study aims to evaluate whether same-day discharge is a viable option compared to conventional fURS under GA. The primary objectives are to assess the outcomes, including efficacy, stone-free rate (SFR), pain management, and complication rates, in the context of same-day discharge. Additionally, this study seeks to identify patient and kidney stone characteristics that may influence the suitability of one-day fURS under SA. Outcomes will be measured using the Dindo–Clavien (D-C) classification and Visual Analog Scale (VAS) scores post-procedure.

## 1. Introduction

In contemporary urological practice, nephrolithiasis, commonly known as kidney stone disease, constitutes a considerable portion of routine clinical encounters and continues to represent a prevalent etiology of global morbidity. Its incidence and prevalence persist as significant clinical challenges, requiring ongoing exploration and management strategies to reduce its impact on patient health and well-being [1]. Renal stone formation intricately intertwines with systemic health, as evidenced by its association with prevalent metabolic disorders such as Type 2 diabetes mellitus, obesity, dyslipidemia, and hypertension. These conditions contribute to the multifaceted etiology of nephrolithiasis, reflecting the complex interplay between metabolic dysregulation and renal physiology. Concurrently, lifestyle factors and environmental influences significantly modulate stone formation, with sedentary habits, dietary imbalances, and geographical variations amplifying the risk profile.

Since the seminal report by Marshall in 1964 detailing the pioneering use of fURS, significant advancements in technology have propelled this modality to the forefront of urological practice. These developmental milestones have culminated in contemporary flexible ureteroscopes characterized by enhanced maneuverability, improved visualization capabilities, and increased durability, thereby facilitating their widespread clinical acceptance. As a result, fURS is now widely used due to its ease of clinical application, high success rate, and low risk of complications. Such technological evolution has revolutionized the management of various urological conditions, empowering clinicians with a versatile and efficacious tool for diagnostic and therapeutic interventions within the urinary tract [2].

Ureteroscopy stands as a formidable approach for managing urinary stones, offering versatility in addressing calculi within both the ureter and kidney. Consequently, it emerges as a compelling alternative to percutaneous nephrolithotomy, presenting comparable efficacy and reduced invasiveness in the treatment of urolithiasis [3].

The convergence of rising incidence rates, elevated stone recurrence rates, and the expanding utilization of minimally invasive treatments presents a substantial financial and economic challenge for healthcare systems [4].

Given constrained resources, there is mounting pressure for procedures to transition to a day-case basis, bypassing inpatient stays. Consequently, same-day discharge following stone treatment emerges as an appealing option, offering financial advantages, potential efficiency gains for healthcare providers, and heightened patient satisfaction [5].

Current guidelines recommend the procedure of flexible ureteroscopy fURS to be performed under GA, resulting in patients typically remaining admitted in the hospital until the next day [6].

## 2. Materials and Methods

Our main endpoint in this paper is to demonstrate that fURS is suitable for SA, allowing for same-day discharge. We aim to show that this approach yields similar results in terms of efficacy, SFR, and pain management compared to the classic fURS procedure performed under GA.

The secondary aim of this study is to identify specific patterns in patient and kidney stone characteristics that could help categorize individuals as either suitable or unsuitable for fURS under SA. This will be achieved through a detailed analysis of post-procedure outcomes, specifically focusing on the D-C grade [7] and VAS scores. By examining these parameters, this study aims to develop criteria that can guide clinical decision-making and optimize patient selection for outpatient fURS procedures. Key variables considered included kidney stone localization, patient age, body mass index (BMI), diabetes status, kidney stone density, and the number of kidney stones. Preliminary findings indicate that fURS under SA can be safely conducted within a one-day hospitalization period for a significant proportion of patients. Factors such as stone localization, patient age, and BMI were significant predictors of procedural safety.

### 2.1. Selection of Study Patients

Our retrospective study included 401 patients who underwent a total of 414 flexible fURS under SA between January 2020 and December 2023 at our medical center.

Patients were seen at a postoperative follow-up control at 45 days. No patients included in this study was lost to follow-up. All patients were evaluated by anesthesiologists, and we recorded their American Society of Anesthesiologists (ASA) scores [8]. In the outpatient fURS Program, we included only patients with ASA I and ASA II scores. Patients with ASA III were not considered for one-day admission fURS.

All patients provided written consent and met the criteria for outpatient surgery. They followed recommendations to be escorted home and not to stay alone on the first night following surgery, as well as to adhere to post-surgery guidelines [9].

### 2.2. Collection of Clinical and Laboratory Data

In 2020, we created a specific database to systematically document a range of patient-specific variables. This database includes detailed records on patient demographics such as gender and background (urban or rural). Patient BMI, diabetes status, and information regarding the use of preoperative stents, including indications for stent placement and the duration for which the stent was in situ prior to the procedure, were also documented. We also recorded parameters related to kidney stones, such as their size, location, number, density, and overall dimensions. Additional clinical details include the ASA score, the duration of the fURS procedure, and the number of fURS procedures performed per patient.

Furthermore, we documented whether an access sheath was employed during the procedure. Postoperative outcomes were also recorded, including the D-C grade, VAS score, and any need for hospitalization following the procedure. Finally, the SFR was assessed at 45 days post-procedure, as was the need for extracorporeal shock wave lithotripsy (ESWL) if residual kidney stones were still present.

Regarding BMI, the categories were defined as follows: normal weight (18.5–24.99), overweight (25–29.99), and obesity (≥30).

Regarding VAS scores, a reported VAS score of 0 indicates that the patient experiences no pain following the intervention. A VAS score between 1 and 3 indicates a mild level of pain, while a score of 4–6 indicates moderate pain. A score of 7–9 indicates very severe pain, and a score of 10 shows the worst pain possible. It is important to clarify that although the VAS score was completed by patients after the procedure, they were instructed to evaluate the pain they experienced specifically during the procedure itself. This distinction is crucial, as all patients underwent the procedure under SA, as previously mentioned.

Regarding the SFR, stone-free status was defined as the presence of kidney stone fragments measuring less than 3 mm in diameter that are identifiable during the procedure when compared to the guide wire and which are not detectable on subsequent ultrasound imaging or as no remaining identifiable stones. Currently, there is no universally accepted definition for the SFR; therefore, this definition was used in our study to standardize the assessment of outcomes.

Failure of outpatient surgery management was defined as the need for a hospital stay longer than 12 h or the need for admission to the Urology Department or Intensive Care Unit (ICU) within 48 postoperative hours.

### 2.3. Treatment

Out of 401 patients, 391 had a preoperative stent, while 10 did not. The duration of stent placement ranged from 14 to 140 days. The most common indications for stent use were as follows: 88 (22.51%) cases of renal colic, 36 (9.21%) cases of obstructive pyelonephritis, 241 (61.64%) cases for preoperative passive dilatation, and 26 (6.65%) cases where the stent was placed post-semirigid ureteroscopy for ureteral lithiasis. The high frequency of pre-stenting reflects the preferences and practices of our team of urologists, who are committed to minimizing all potential risks associated with the procedure, especially considering the one-day admission and outpatient setting. Pre-stenting was routinely used to ensure optimal conditions for ureteroscopy and to enhance patient safety and outcomes.

Regarding the clinical course, patients are admitted to our department two hours before surgery. Anesthesiology examination and routine blood analysis verification are conducted, followed by obtaining informed consent for anesthesia and surgery. If the anesthesiologist approves SA, ceftriaxone 2 g intravenous is administered one hour before surgery for prophylaxis. The patient is positioned in the lithotomy position. A semirigid 7 Ch Karl Storz ureterorenoscope (Karl Storz Endoscopia SRL, Bucharest, Romania) is introduced into the bladder and the double J stent is extracted if pre-stenting was performed. Using a Terumo nitinol hydrophilic 0.035 Ch guidewire (Terumo Europe, Antwerp, Belgium), access to the ureteral orifice is facilitated, and the guidewire is advanced up to the renal pelvis. The ureteral caliber is evaluated, and any small stones present in the ureter are removed. A ureteral access sheath, 12 Ch/10 Ch, Cook/ClearPetra Wellead^®^ (Well Lead Medical Co., Ltd., Guangzhou, China), is inserted over the guidewire and advanced up to the ureteropelvic junction under direct visualization from the bladder. An access sheath (10 Ch or 12 Ch) was used in all patients except for 20, where it was deemed unnecessary. The decision to use an access sheath was based on the preferences of the surgeons and aimed to standardize the procedure across all patients, thereby minimizing bias and ensuring consistency in our approach.

Upon confirming the correct placement of the access sheath, a flexible ureteroscope is advanced to the renal pelvis using the guidewire, allowing for thorough evaluation of the pelvis, calyces, and stones. Stone fragmentation is achieved using holmium/yttrium–aluminum–garnet (Ho:YAG) laser energy (Hyper Photonics SRL, Monza, Italy) with a 365 μm laser fiber. A stone extractor, NGage Nitinol (Cook Medical LLC, Bloomington, IN, USA), is utilized as needed. Upon completion of fragmentation and confirmation of stone-free status, a double J stent (6 Ch) is placed to ensure proper urinary drainage and prevent ureteral obstruction.

Procedures were performed using various ureteroscopes: Karl Storz Reusable Optical Flexible Ureteroscope 7.5 Ch in 62 cases—14.73%, Seesheen Reusable Digital Flexible Ureteroscope 9.5 Ch (Zhuhai Seesheen Medical Technology, Zhuhai, China) in 115 cases—27.78%, HugeMed Digital Flexible Ureteroscope Single Use (HugeMed, Guangzhou, China) 7.5 Ch in 145 cases—35.02%, and HugeMed Digital Flexible Ureteroscope Single Use 9 Ch in 92 cases—22.22%. All procedures utilized a Terumo Nitinol Hydrophilic Guidewire. The choice of instruments depended on the availability in our department and the surgeon’s preference. We also aimed to homogenize all patient groups regarding the instrument of choice.

To counter precision loss due to kidney movement under SA, patients were instructed to hold their breath for approximately 10 s at specific moments during the laser fragmentation procedure, particularly when the flexible ureteroscope approached the stone and laser energy was being delivered, requiring maximum precision. This measure was adopted to minimize sudden movements, enhancing treatment accuracy and contributing to the overall SFR.

Kidney stones were treated using a Ho:YAG laser with 365 μm reusable fibers and NGage Nitinol Stone Extractor. A double J 6 Ch ureteral stent was placed in all patients after surgery. Postoperatively, all patients received analgesia and were discharged within 4 to 5 h after the surgical procedure, following a joint decision with the anesthesiologist.

In cases where complete elimination of the kidney stone could not be achieved, whether due to patient-related complications or an extended procedural duration deemed unsafe, a second procedure was performed. This subsequent procedure was scheduled either after the resolution of the symptoms that led to the initial procedure’s conclusion or after a variable period ranging from 10 to 15 days.

The SFR was assessed 45 days postoperatively using ultrasound or computer tomography (CT) imaging. In cases where residual kidney stones were detected, ESWL was employed for further treatment. Specifically, ESWL was used for patients who did not meet the criteria to qualify as “stone-free”, meaning those with stones detectable on ultrasound or CT imaging. This approach was taken to address residual stones that remained after the initial procedure and to achieve optimal stone clearance.

### 2.4. Statistical Analysis

We considered factors such as the size, density, and number of kidney stones, their location within the kidney, the duration of the fURS procedure, the number of fURS procedures each patient underwent, the presence of an access sheath, patient age, and lifestyle indicators such as BMI and whether or not they have diabetes. We evaluated the effects of these factors on the D-C grade and the VAS score reported by the patients. We aimed to assess to what extent patients with certain characteristics of these factors are more likely to report a higher D-C grade or a higher VAS score following the fURS intervention. Our results are expressed in terms of relative risk. Statistical analyses were performed using Microsoft Excel for Mac, Version 16.82 (Microsoft Corporation, Redmond, WA, USA).

## 3. Results

### 3.1. Patient Characteristics

The demographic and clinical characteristics of the 401 study participants are presented in Table 1.

Out of the 401 patients, 282 presented a solitary kidney stone, whereas 119 presented with multiple kidney stones. Among the latter group, 93 exhibited stones in disparate anatomical locations, whereas 26 had multiple stones confined to a single anatomical location. The locations of kidney stones were classified into five distinct categories. To facilitate a comprehensive statistical analysis, patients with multiple localizations were categorized based on the most challenging localization of a single kidney stone. For patients with a single kidney stone, or those with multiple stones all situated in the same anatomical region, they were assigned to the category corresponding to that specific localization. The categorization criteria were as follows:

Coralliform: patients with staghorn calculi occupying a significant portion of the renal pelvis and branching into several or all calyces;

Lower calyx: patients with at least one kidney stone located in the lower calyx, regardless of the location of other kidney stones;

Middle calyx: patients with at least one kidney stone located in the middle calyx, regardless of the location of other kidney stones, with no stones located in the lower calyx;

Upper calyx: patients with at least one stone located in the upper calyx, regardless of the location of other kidney stones, with no stones located in the lower or middle calyces;

Renal pelvis: patients with stones located exclusively in the renal pelvis, with no stones in the lower, middle, or upper calyces.

**Figure 1:**
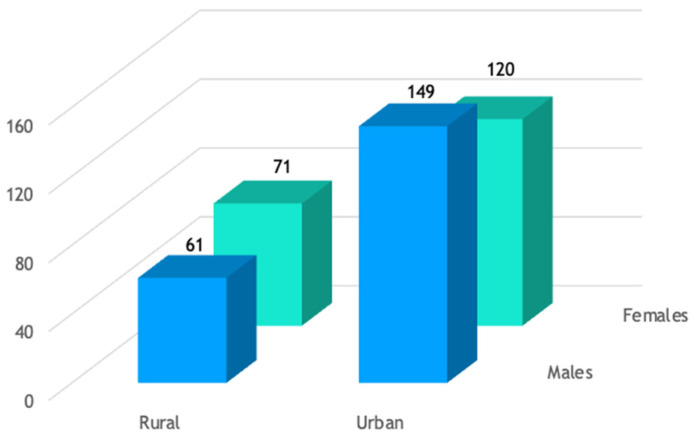
Patient distribution by gender and background.

**Figure 2:**
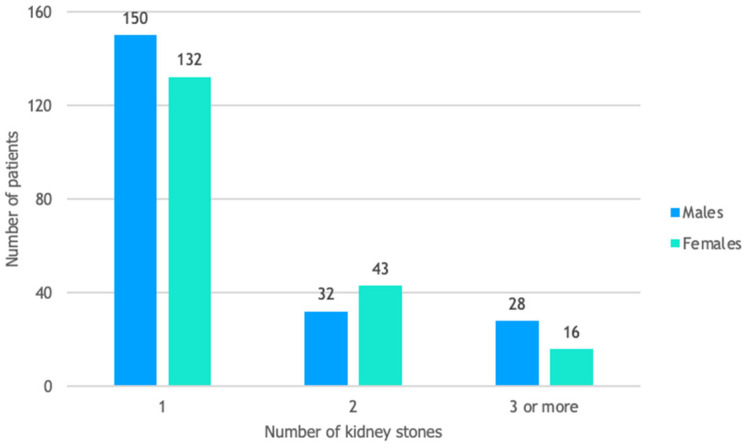
Patient distribution by gender and number of kidney stones.

The distribution of patients by gender and background is shown in Figure 1. Most patients in our study were from urban areas, accounting for 67% of the cohort. Figure 2 illustrates the distribution of patients by gender and the number of kidney stones. Among the study group, 70% had a solitary kidney stone, 19% had two kidney stones, and 11% (44 out of 401) had three or more kidney stones.

### 3.2. VAS Score and Number of Kidney Stones

**Figure 3:**
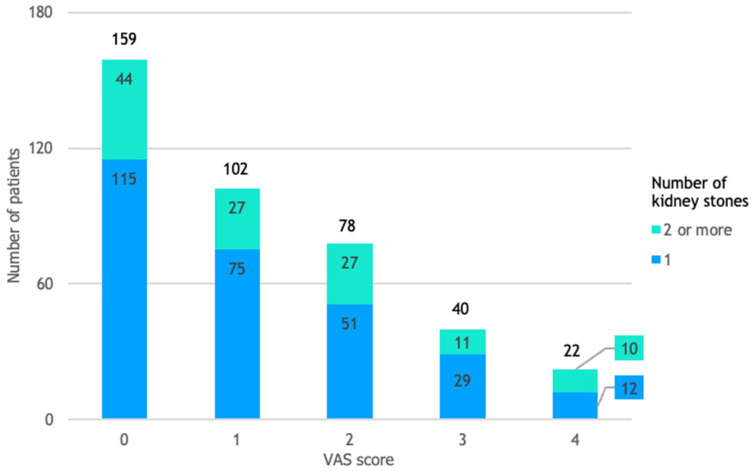
Patient distribution by VAS score and number of kidney stones. The relative risk was 1.97, indicating that patients with multiple kidney stones (2 or more) were 97% more likely to report moderate pain, rather than mild or no pain, compared to those with a single kidney stone.

None of the patients reported a VAS score above 4. Out of 401 patients, 159 (40%) reported no pain, 220 (55%) reported mild pain, and 22 (5%) reported moderate pain during the fURS procedure, as shown in Figure 3.

### 3.3. VAS Score and Size of Kidney Stones

**Figure 4:**
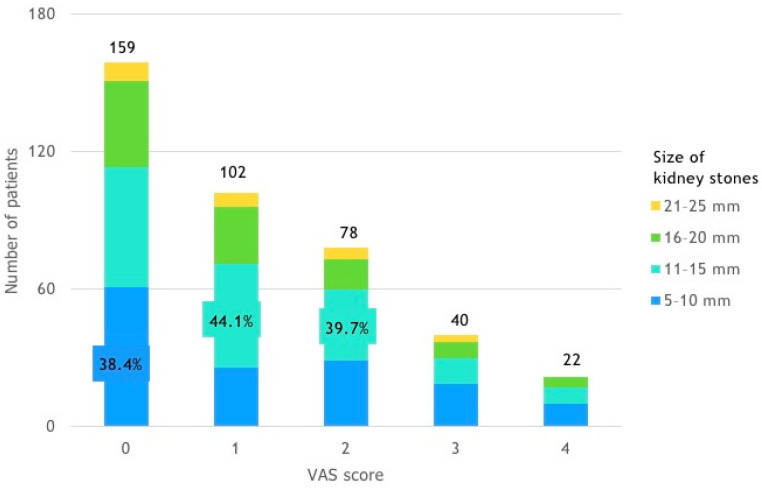
Patient distribution by VAS score and size of kidney stones.

Patients with a VAS score of 0 mostly had smaller stones, with 38.4% having stones between 5 and 10 mm. In contrast, patients with VAS scores of 1 and 2 more frequently had larger stones, with 44.1% and 39.7% of these patients, respectively, having stones between 11 and 15 mm (Figure 4).

### 3.4. VAS Score and Location of Kidney Stones

**Figure 5:**
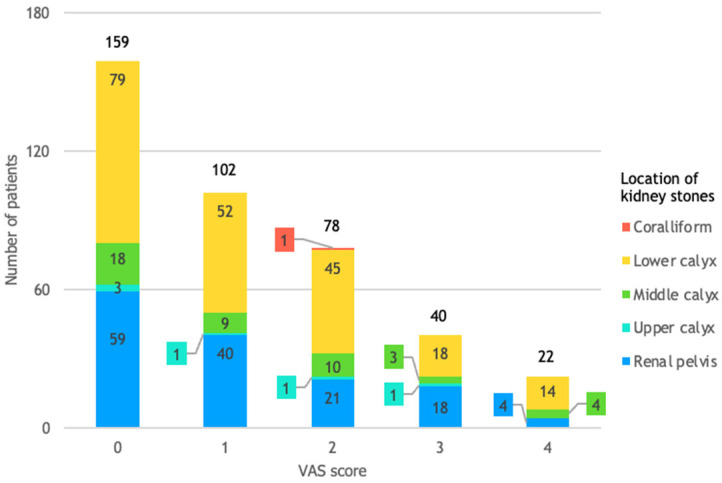
Patient distribution by VAS score and location of kidney stones.

Figure 5 shows that 82% of the 22 patients with a VAS score of 4, indicating moderate pain, had kidney stones in the lower or middle calyx. It also illustrates that patients with stones in the lower calyx are more than twice as likely (relative risk 2.39) to experience moderate pain compared to those with stones in the renal pelvis. Furthermore, patients with stones in the middle calyx are more than three times as likely (relative risk 3.23) to report moderate pain compared to those with stones in the renal pelvis.

### 3.5. VAS Score and fURS Duration

**Figure 6:**
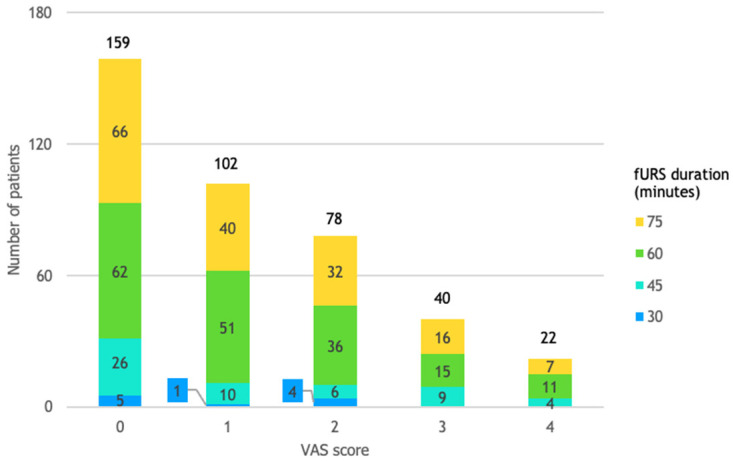
Patient distribution by VAS score and fURS duration.

All patients that reported VAS scores of 3 or 4 underwent procedures of at least 45 min. All procedures that had a duration of 30 min were followed by mild or no pain (Figure 6).

### 3.6. VAS Score and BMI

The split of patients according to BMI and VAS score is detailed in Table 2. A total of 320 out of 401 patients (80%) were overweight or obese.

### 3.7. VAS Score and Diabetes

Table 3 details the distribution of patients by diabetes condition and reported VAS score. The relative risk of 1.40 indicates that patients with diabetes are 40% more likely to experience higher levels of pain compared to those without diabetes.

### 3.8. D-C Grade and Number of Kidney Stones

**Figure 7:**
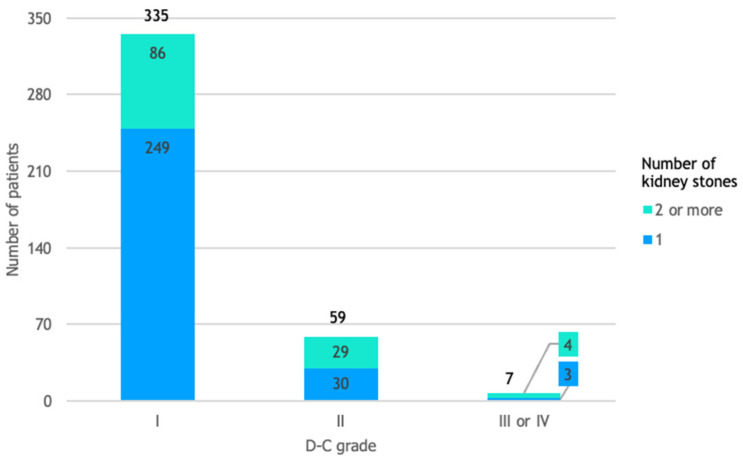
Patient distribution by D-C grade and number of kidney stones. This figure assesses the relative risk of experiencing complications classified as D-C grade II, III, or IV, rather than D-C grade I, in patients with 2 or more kidney stones compared to those with only 1 kidney stone. The relative risk of 2.37 indicates that patients with more than 1 kidney stone were over twice as likely to experience more serious complications compared to those with a single kidney stone.

A total of 335 out of 401 patients (84%) experienced mild or no complications following the fURS procedure (D-C grade I), while the other 66 patients (16%) experienced D-C grade II, III, or IV complications, as detailed by Figure 7.

### 3.9. D-C Grade and Size of Kidney Stones

**Figure 8:**
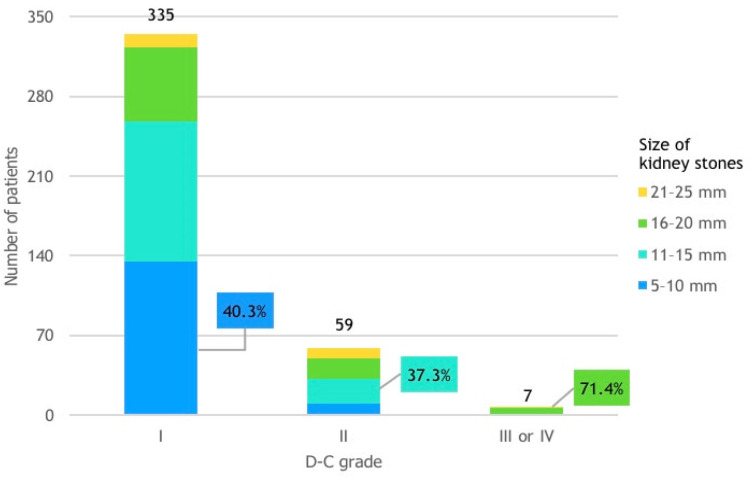
Patient distribution by D-C grade and size of kidney stones. Patients with 11–15 mm kidney stones were over twice as likely to experience complications compared to those with 5–10 mm stones. Those with 16–20 mm stones were nearly 4 times more likely to have complications, and patients with 21–25 mm stones were over 6 times more likely to experience complications compared to those with 5–10 mm stones.

The majority of patients in D-C grade I exhibited smaller kidney stones, with 40.3% of the patients presenting stones between 5 and 10 mm. Conversely, in D-C grade II, larger kidney stones were more prevalent, with 37.3% of the patients having stones sized between 11 and 15 mm. Lastly, in D-C grade III or IV, relatively large kidney stones were predominant, with 71.4% of the patients presenting with stones sized between 16 and 20 mm (Figure 8).

### 3.10. D-C Grade and Location of Kidney Stones

**Figure 9:**
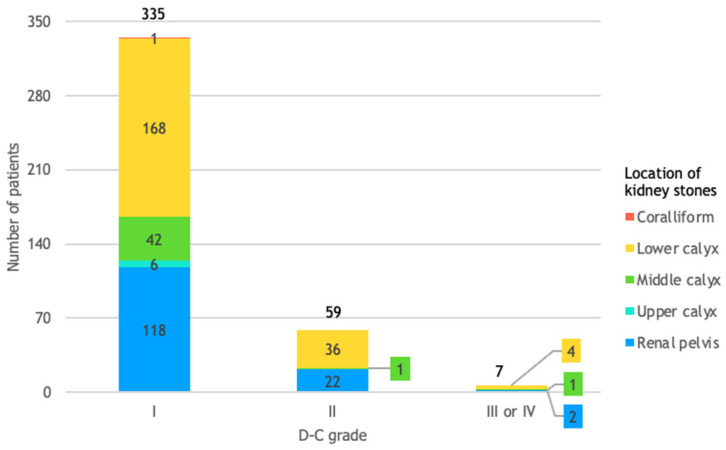
Patient distribution by D-C grade and location of kidney stones. This figure highlights the relative risk of experiencing complications classified as D-C grade II, III, or IV, for patients with kidney stones in the lower calyx compared to those with stones in the middle calyx. The relative risk of 4.23 indicates that patients with stones in the lower calyx were over 4 times more likely to experience more serious complications than those with stones in the middle calyx.

Figure 9 shows that 5 out of the 7 patients (71.4%) in D-C grade III or IV had at least one kidney stone in the lower or middle calyx, and 37 out of the 59 patients (62.7%) in D-C grade II had at least one kidney stone in the lower or middle calyx.

### 3.11. D-C Grade and fURS Duration

**Figure 10:**
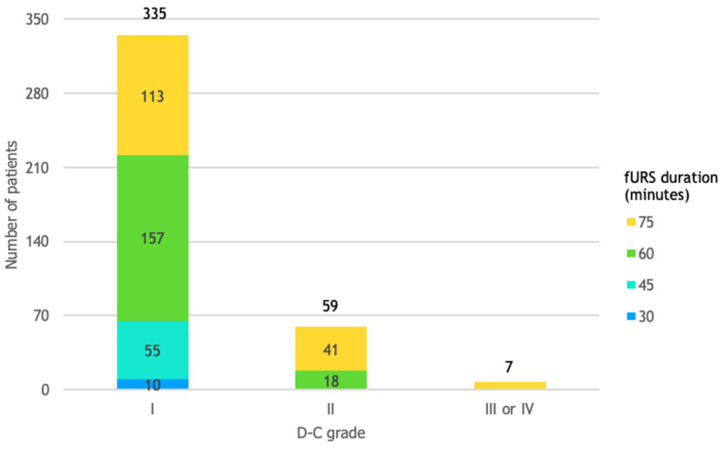
Patient distribution by D-C grade and fURS duration. This figure shows that patients undergoing 75 min procedures were almost 3 times more likely (relative risk of 2.90) to experience complications classified as D-C grade II, III, or IV, rather than D-C grade I, compared to patients undergoing 60 min procedures.

All procedures classified as D-C grade III or IV had a duration of 75 min, while procedures classified as D-C grade II had a duration of either 60 min or 75 min. All procedures that had a duration of 30 min or 45 min were followed by mild or no complications (Figure 10).

### 3.12. D-C Grade and BMI

The split of patients according to BMI and the D-C grade is detailed in Table 4. Table 4 evaluates the relative risk of experiencing complications classified as D-C grade II, III, or IV, rather than D-C grade I, for overweight or obese patients compared to those of normal weight. The relative risk of 1.14 indicates that overweight or obese patients were 14% more likely to experience more serious complications than normal weight patients.

### 3.13. D-C Grade and Diabetes

A total of 55 out of 401 patients (14%) had diabetes. The split of patients according to diabetes status and D-C complication classification is detailed in Table 5.

### 3.14. Hospitalization Rate Post-Procedure

Out of the 401 patients, 7 (5 males and 2 females) required hospitalization, thus meeting the criteria for failure of outpatient surgical management. Among these, three males presented with solitary kidney stones (average size: 20 mm; average density: 775 Hounsfield Units (HUs)), two patients had two stones each (average size: 15.5 mm; average density: 1051.5 HUs), one patient had three stones (average size: 12 mm; average density: 1070 HUs), and one patient had six stones (average size: 6.3 mm; average density: 1708 HUs).

There are many factors attesting the complexity of these seven instances. More precisely, all seven patients that required hospitalization had undergone procedures of 75 min. Moreover, the D-C complication grades of these seven instances were either III or IV. Additionally, five out of these seven patients had at least one kidney stone located in the middle or lower calyces. Finally, five out of these seven patients were older than 65 years.

However, in our study cohort, the majority of patients, specifically 394 individuals (98%) out of the total 401 participants, did not necessitate hospitalization post-surgery. Among these patients, 384 had received a preoperative ureteral stent, whereas 10 did not. Within the latter group, eight patients presented with relatively small kidney stones (less than 10 mm), one had a kidney stone measuring 12 mm, and another patient had a kidney stone measuring 20 mm. The average stone density among these 10 patients was recorded at 1269.9 HUs. All 10 patients exhibited a D-C grade I. It is noteworthy that only one participant, a 58-year-old male, underwent the procedure with the aid of an access sheath.

### 3.15. Overall SFR Outcomes

The SFR assessed at 45 postoperative days via ultrasound or CT scan was nearly perfect in the study cohort. Among the 401 patients included, only 2 individuals still presented with kidney stones. The duration of the fURS procedure for these cases was 75 min, indicating the complexity of these instances. Subsequently, both patients underwent an ESWL procedure.

Thirteen patients required two procedures; however, hospitalization was not necessary, and follow-up ultrasound or CT scans after 45 days demonstrated the absence of kidney stones. Among these thirteen patients, eight had a solitary kidney stone located in the renal pelvis, with an average size of 21.25 mm and an average stone density of 1161.5 HUs. The remaining five patients had multiple kidney stones, including at least one in the lower calyx, which increases the procedural difficulty. These multiple stone cases had an average size of 15 mm and an average stone density of 1112.2 HU. In all 13 cases requiring two procedures, at least one of the procedures lasted either 75 or 60 min, further highlighting the complexity of these scenarios.

### 3.16. Summary

The fURS procedure performed under SA is a safe and efficient intervention, as proven by the high SFR (399 out of 401; 99.5%) and low hospitalization rate (7 out of 401; 1.7%). Nevertheless, we summarized our results (Table 6) to identify specific risk groups of patients who were more likely to experience pain during the procedure as measured by the VAS score and who were also more likely to experience complications following the fURS procedure, as assessed by the D-C grade. Based on these findings, we developed a risk assessment scale named “SAFER” (Safety Assessment of Flexible Endoscopic Risk) to evaluate the safety of performing fURS under SA for same-day admission. The SAFER scale aims to systematically identify and categorize patients as suitable or unsuitable for this procedure based on specific patient and kidney stone characteristics. This scale is currently utilized in our department to guide risk assessment for patients prior to the fURS procedure and has shown satisfactory results.

Additionally, we aimed to provide insights into the level of pain reported by patients, evaluated using the VAS score, and to correlate this pain level with various patient and kidney stone characteristics.

Moreover, our study allowed us to define four major risk categories for patients. Specifically, we determined that each patient had either a low, moderate, high, or very high risk of developing complications and/or experiencing pain following the fURS intervention. This categorization is illustrated in the “SAFER” scale patient classification (Table 7). The size and location of the kidney stone(s) were two of the most important factors indicating this risk. Additionally, the number of kidney stones, the duration of the fURS procedure, the presence of diabetes, and the patient’s BMI were factors that influenced the risk, as detailed in Table 7.

In our study of 401 patients, several factors were identified as risk factors for complications and pain:

- Number of kidney stones: Patients with more than one kidney stone are 2.37 times more likely to experience moderate or severe complications and 1.97 times more likely to report moderate pain compared to those with a solitary stone.
- Size of kidney stones: Larger stones increase complication risks. Patients with stones > 20 mm are over 6 times more likely to experience moderate or severe complications compared to those with stones < 10 mm.
- Location of kidney stones: Stones in the lower calyx are 4.23 times more likely to cause moderate or severe complications and 2.39 times more likely to result in moderate pain compared to stones in the renal pelvis. Stones in the middle calyx are 3.23 times more likely to cause moderate pain compared to those in the renal pelvis. However, treatment outcomes for stones in the lower calyx were consistent, regardless of the final treatment location.
- Duration of fURS procedure: A procedure lasting 75 min is nearly 3 times more likely to result in moderate or severe complications than a 60 min procedure.
- Diabetes: Diabetic patients are 40% more likely to report moderate pain compared to non-diabetic patients.
- BMI: Overweight or obese patients are 14% more likely to experience moderate or severe complications compared to those of normal weight.

## 4. Discussion

### 4.1. Cross-Analysis

Ambulatory surgery is promoted globally for its economic benefits [9]. Nephrolithiasis, though potentially affecting all ages, is most prevalent in individuals aged 20–49 years, highlighting the need for targeted screening and early intervention. This age peak highlights the importance of preventive measures in early adulthood. Gender differences are also notable, with males more susceptible to stone formation than females, likely due to hormonal, anatomical, and behavioral factors. In summary, nephrolithiasis involves a complex interplay of metabolic, lifestyle, and demographic factors. Understanding these relationships is crucial for developing effective preventive and therapeutic strategies to address renal stone disease worldwide [10].

Renal stones are common in individuals with obesity and diabetes, illustrating the link between metabolic issues and stone formation. With around 50% of patients experiencing recurrence within 5 years, effective long-term management, including metabolic control, dietary changes, and hydration, is crucial [10]. Global warming is projected to increase renal stone prevalence from 40% to 56% by 2050 due to higher dehydration and altered precipitation affecting dietary habits and stone risk [11]. Additionally, obesity, including its rise in pediatric populations, is a significant factor in kidney stone formation [12].

With the rising incidence of nephrolithiasis and a shift towards day-case procedures, there is an urgent need to manage costs and improve patient satisfaction. In our practice, ESWL and ureteroscopic lithotripsy (URSL) are the most common treatments [13]. Recent advancements in retrograde intrarenal surgery (RIRS) show a cumulative stone-free rate of 91% for stones > 2 cm, with an average of 1.45 procedures per patient and 4.5% of complications at Clavien grade 3 or higher [14]. The use of digital scopes has further reduced operation times by enhancing image quality [15,16].

While GA is commonly used for most interventions, the guidelines do not make a specific recommendation for the type of anesthesia. Local or spinal anesthesia may also be employed. Regarding the D-C grade post-procedure, no significant difference is observed in the incidence of grades 1, 2, or 3 between procedures performed under SA and those performed under GA [17]. fURS is a preferred minimally invasive method for treating upper urinary tract stones due to its safety, noninvasiveness, and ease of manipulation. It is often performed as a day-case or outpatient procedure [18].

There are only two studies in the literature analyzing the results of fURS in a true outpatient setting, and both are retrospective reviews [14]. Preoperative stenting before fURS for passive dilation of the ureter remains under debate. However, the results of published meta-analyses may support the practice of preoperative stenting [19]. Routine postoperative double J stenting significantly contributes to postoperative pain relief and facilitates rehabilitation, particularly in outpatient surgery. Overall stent insertion rates in the Clinical Research Office of Endourological Society (CROES) data on 11,885 cases were 81.5%. Their data comprised 82% (*n* = 9681) ureteric stones, of which 46% were in the distal ureter and had a substantially lower postoperative stent insertion rate of 55% [19].

Although fURS showed no advantage over shock wave lithotripsy (SWL) in treating lower pole stones ≤ 1 cm in a randomized controlled trial conducted by the Lower Pole Study Group, fURS remains a valuable option for certain patients and clinical scenarios [20]. More recent studies have demonstrated advantages in favor of fURS over alternative treatments, suggesting its efficacy and benefits in managing lower pole stones [21]. In 2012, El-Nahas et al. analyzed a matched-pair group of patients with 10–20 mm lower pole stones treated with fURS or SWL. The study found that fURS had a statistically significantly higher SFR (86.5% vs. 67.7% for SWL) and a lower retreatment rate (8% vs. 60% for the SWL group) [22]. According to the literature, complications after ureteroscopic surgery are rare, with a global morbidity rate typically ranging between 5% and 10% [19].

The overall complication rate after fURS is reported to be between 9% and 25% [23]. The CROES URS global study has identified bleeding, fever, urinary tract infections (UTIs), and sepsis as common postoperative complications following fURS. With the increasing incidence and high recurrence rates of urinary stones, as well as the annual increase in patients undergoing fURS, it is expected that the instances of such complications will also rise [24].

During the procedure, the pain experienced by the patient is primarily caused by sudden increases in intrarenal pressure due to the irrigation flow. Patient feedback allows the surgeon to monitor and adjust the intrarenal pressure by reducing the irrigation flow, thereby limiting the pain felt during and especially after the procedure. This real-time feedback from the patient is not possible under GA. In our opinion, this is one of the main advantages of SA, as many complications during and post-procedure are associated with elevated intrarenal pressure.

Under GA, the controllability of kidney movement is better due to the artificial control of breathing, while under SA, the patient maintains spontaneous breathing, resulting in lower controllability of movement [25]. However, a recent meta-analysis did not show this outcome, indicating that the results are similar in terms of SFR for both GA and SA [17]. To facilitate the procedure for patients under SA, the patient can be asked to cooperate with the surgeon, such as by deep breathing and maintaining a fixed position. Karabulut et al. [26] published similar results, showing that the same SFR efficacy is obtained when patients under SA are asked to hold their breath, comparable to patients under GA where the anesthesiologist controls the breathing. These results are consistent with our own findings. In our study, if needed, patients were asked to hold their breath for 10–15 s to facilitate greater surgical precision. As shown before, the SFR in our study was nearly perfect, demonstrating that this method is highly effective.

To our knowledge, this is the first study to measure the VAS score during the procedure, rather than the usual post-procedure assessment. This approach was implemented to evaluate whether the fURS procedure, regardless of its complexity, is suitable for SA. By assessing pain in real time during the procedure, we aimed to provide a more accurate evaluation of patient discomfort and the feasibility of SA in various clinical scenarios. As mentioned previously, it is crucial to note that although the VAS score was completed by patients post-procedure, they were specifically instructed to assess the pain experienced during the procedure, not after. This distinction ensures that the VAS score accurately reflects the patient’s discomfort related to the procedure itself, rather than postoperative pain. In this paper, the choice of treatment for outpatients was based on the recommendations outlined by the EAU guidelines [6]. According to these guidelines, stones larger than 20 mm are typically managed with percutaneous nephrolithotomy, while stones smaller than 20 mm are generally treated with FURS or ESWL.

### 4.2. Study Limitations

This study acknowledges several limitations that may impact the interpretation of the results. First, the retrospective nature of the study design introduces potential biases related to the selection of patients and the collection of data. Retrospective studies are inherently limited by the availability and accuracy of historical data, which can affect the reliability of the findings.

Second, the heterogeneity of the patient population and the variability in methods used are notable limitations. Specifically, variations in the caliber of the ureteroscopes and the ureteral access sheaths may influence the VAS scores. These differences can affect pain perception and management outcomes, and this study’s results may not fully account for these variations.

Additionally, pre-stenting was used routinely across the study cohort due to surgeon preference, which could have influenced the outcomes and may not reflect the practices of other institutions. Similarly, the universal application of the ureteral access sheath was based on the surgeons’ preferences to standardize the procedure.

These factors should be considered when interpreting this study’s findings and their generalizability. Future prospective studies with more homogeneous populations and standardized methodologies are needed to validate these results and provide more robust conclusions.

Moreover, given that all patients were discharged on the same day of the procedure, it is possible that some patients experienced minor complications but did not report them, either because the complications resolved spontaneously or because the patients did not consider them relevant. This introduced subjectivity into the assessment process, as it relied on patient reports rather than on the objective day-by-day assessment made by medical professionals, as would occur if the patient stayed in the hospital for several days.

## 5. Conclusions

In our opinion, fURS under SA is a safe procedure that can be performed by experienced surgeons in outpatient surgery departments for the treatment of kidney stones. Most kidney stones can be effectively treated using fURS in a single day, utilizing flexible digital ureteroscopes with minimal postoperative complications. Additionally, we have observed that fURS is a favorable treatment option for multiple kidney stones or stones in different positions within the kidney in the outpatient setting. Adopting this approach for kidney stone treatment results in reduced overall costs and decreased morbidity. We hope the ”SAFER” scale will be adopted by clinicians worldwide as a standardized tool to evaluate patient suitability for fURS under SA in outpatient clinical settings. By providing a reliable and objective measure of risk, the “SAFER” scale aims to enhance clinical decision-making, ensuring that patients receive the most appropriate and safe treatment for kidney stones. This scale has the potential to streamline patient selection processes, optimize treatment outcomes, and contribute to the advancement of minimally invasive urological practices.

## Figures and Tables

**Table 1:** Demographic and clinical characteristics of study subjects.

Patient Characteristics		*n* = 401
Gender	F	191
	M	210
Age (years) *	Min	18
	Max	84
		50.6 ± 14.1
Kidney involved	Right	185
	Left	216
Kidney stone size (mm) *	Min	5
	Max	25
		13.7 ± 4.8
Stone location	Renal pelvis	142
	Upper calyx	6
	Middle calyx	44
	Lower calyx	208
	Coralliform	1
Number of kidney stones	1	282
	2	75
	≥3	44
Kidney stone density (Hounsfield Units) *	Min	604
	Max	1733
		1188.7 ± 334.1
Preoperative stent	Yes	391
	No	10
Stent duration (days)	0	10
	14	195
	20 or 21	173
	>21	23
Number of fURS procedures	1	388
	2	13
SFR	Yes	399
	No	2
D-C complication grade	I	335
	II	59
	III or IV	7
Procedure duration (minutes)	30	10
	45	55
	60	175
	75	161
Access sheath	Yes	381
	No	20
ASA score	1	53
	2	348
Need for ESWL post-op	Yes	3
	No	398
BMI	Normal weight	81
	Overweight	198
	Obesity	122
Diabetes	Yes	55
	No	346
VAS score	0	159
	1	102
	2	78
	3	40
	4	22

* Values are expressed as mean ± standard deviation.

**Table 2:** Patient distribution by VAS score and BMI.

		VAS Score	
		Moderate Pain (Score 4)	Mild or No Pain (Score 0, 1–3)
BMI	Normal weight	7	74
	Overweight or obese	15	305

**Table 3:** Patient distribution by VAS score and diabetes status.

		VAS Score	
		Moderate Pain (Score 4)	Mild or No Pain (Score 0, 1–3)
Diabetes	Yes	4	51
	No	18	328

**Table 4:** Patient distribution by D-C grade and BMI.

		D-C Grade	
		I (Mild or No Complications)	II, III or IV (Moderate or Severe Complications)
BMI	Normal weight	69	12
	Overweight or obese	266	54

**Table 5:** Patient distribution by D-C grade and diabetes status.

		D-C Grade	
		I (Mild or No Complications)	II, III or IV (Moderate or Severe Complications)
Diabetes	Yes	46	9
	No	289	57

**Table 6:** Relative risk summary scale of fURS—”SAFER” scale (Safety Assessment of Flexible Endoscopic Risk).

		D-C Grade		VAS Score	
		I (Mild or No Complications)	II, III, or IV (Moderate or Severe Complications)	Mild or No Pain (Score 0, 1–3)	Moderate Pain (Score 4)
Number of stones	1	Reference category		Reference category	
	≥2		2.37		1.97
Stone size	5–10 mm	Reference category			-
	11–15 mm		2.28		-
	16–20 mm		3.79		-
	21–25 mm		6.59		-
Stone location	Renal pelvis		-	Reference category	
	Upper calyx		-		-
	Middle calyx	Reference category			3.23
	Lower calyx		4.23		2.39
	Coralliform		-		-
Procedure duration	30 min		-		-
	45 min		-		-
	60 min	Reference category			-
	75 min		2.90		-
Diabetes	No		-	Reference category	
	Yes		-		1.40
BMI	Normal weight	Reference category			-
	Overweight or obese		1.14		-

**Table 7:** “SAFER” scale patient classification.

Stone Location	Stones Number	Procedure Duration (min)	Diabetes (Y/N)	BMI	Stone Size			
					<10 mm	11–15 mm	16–20 mm	>20 mm
Renal pelvis	1	≤60	N	<25	Low	Low	Low	Moderate
				≥25	Low	Low	Low	Moderate
			Y	<25	Low	Low	Low	Moderate
				≥25	Low	Low	Low	Moderate
		>60	N	<25	Low	Low	Moderate	Moderate
				≥25	Low	Low	Moderate	Moderate
			Y	<25	Low	Low	Moderate	Moderate
				≥25	Low	Low	Moderate	Moderate
	≥2	≤60	N	<25	Low	Moderate	Moderate	High
				≥25	Low	Moderate	Moderate	High
			Y	<25	Low	Moderate	Moderate	High
				≥25	Low	Moderate	Moderate	High
		>60	N	<25	Low	Moderate	Moderate	High
				≥25	Low	Moderate	Moderate	High
			Y	<25	Low	Moderate	Moderate	High
				≥25	Low	Moderate	Moderate	High
Upper or middle calyx	1	≤60	N	<25	Low	Moderate	High	High
				≥25	Low	Moderate	High	High
			Y	<25	Low	Moderate	High	High
				≥25	Low	Moderate	High	High
		>60	N	<25	Low	Moderate	High	High
				≥25	Low	Moderate	High	High
			Y	<25	Low	Moderate	High	High
				≥25	Low	Moderate	High	High
	≥2	≤60	N	<25	Moderate	Moderate	High	High
				≥25	Moderate	Moderate	High	High
			Y	<25	Moderate	Moderate	High	High
				≥25	Moderate	Moderate	High	High
		>60	N	<25	Moderate	Moderate	High	Very high
				≥25	Moderate	Moderate	High	Very high
			Y	<25	Moderate	Moderate	High	Very high
				≥25	Moderate	Moderate	High	Very high
Lower calyx	1	≤60	N	<25	High	High	High	Very high
				≥25	High	High	High	Very high
			Y	<25	High	High	High	Very high
				≥25	High	High	High	Very high
		>60	N	<25	High	High	Very high	Very high
				≥25	High	High	Very high	Very high
			Y	<25	High	High	Very high	Very high
				≥25	High	High	Very high	Very high
	≥2	≤60	N	<25	Very high	Very high	Very high	Very high
				≥25	Very high	Very high	Very high	Very high
			Y	<25	Very high	Very high	Very high	Very high
				≥25	Very high	Very high	Very high	Very high
		>60	N	<25	Very high	Very high	Very high	Very high
				≥25	Very high	Very high	Very high	Very high
			Y	<25	Very high	Very high	Very high	Very high
				≥25	Very high	Very high	Very high	Very high

## Data Availability

The datasets produced or analyzed during the present study are available from the corresponding author upon reasonable request.

## Abbreviations

fURS	flexible ureteroscopy
GA	general anesthesia
SA	spinal anesthesia
SFR	stone-free rate
D-C	Dindo–Clavien
VAS	Visual Analog Scale
BMI	body mass index
ASA	American Society of Anesthesiologists
ESWL	extracorporeal shock wave lithotripsy
ICU	Intensive Care Unit
Ho: YAG	holmium/yttrium–aluminum–garnet
CT	computer tomography
HUs	Hounsfield Units
ACT	adjustable continence therapy
URSL	ureteroscopic lithotripsy
RIRS	retrograde intrarenal surgery
CROES	Clinical Research Office of Endourological Society
SWL	shock wave lithotripsy
UTI(s)	urinary tract infections
URS	ureterorenoscopy
